# Impact of Indian Total Sanitation Campaign on Latrine Coverage and Use: A Cross-Sectional Study in Orissa Three Years following Programme Implementation

**DOI:** 10.1371/journal.pone.0071438

**Published:** 2013-08-21

**Authors:** Sharmani Barnard, Parimita Routray, Fiona Majorin, Rachel Peletz, Sophie Boisson, Antara Sinha, Thomas Clasen

**Affiliations:** Department of Disease Control, Faculty of Infectious and Tropical Diseases, London School of Hygiene and Tropical Medicine, London, United Kingdom; Kenya Medical Research Institute - Wellcome Trust Research Programme, Kenya

## Abstract

**Background:**

Faced with a massive shortfall in meeting sanitation targets, some governments have implemented campaigns that use subsidies focused on latrine construction to overcome income constraints and rapidly expand coverage. In settings like rural India where open defecation is common, this may result in sub-optimal compliance (use), thereby continuing to leave the population exposed to human excreta.

**Methods:**

We conducted a cross-sectional study to investigate latrine coverage and use among 20 villages (447 households, 1933 individuals) in Orissa, India where the Government of India’s Total Sanitation Campaign had been implemented at least three years previously. We defined coverage as the proportion of households that had a latrine; for use we identified the proportion of households with at least one reported user and among those, the extent of reported use by each member of the household.

**Results:**

Mean latrine coverage among the villages was 72% (compared to <10% in comparable villages in the same district where the Total Sanitation Campaign had not yet been implemented), though three of the villages had less than 50% coverage. Among these households with latrines, more than a third (39%) were not being used by any member of the household. Well over a third (37%) of the members of households with latrines reported never defecating in their latrines. Less than half (47%) of the members of such households reported using their latrines at all times for defecation. Combined with the 28% of households that did not have latrines, it appears that most defecation events in these communities are still practiced in the open.

**Conclusion:**

A large-scale campaign to implement sanitation has achieved substantial gains in latrine coverage in this population. Nevertheless, gaps in coverage and widespread continuation of open defecation will result in continued exposure to human excreta, reducing the potential for health gains.

## Background

An estimated 2.5 billion people lack access to improved facilities for the disposal of human excreta, such as a basic pit latrine [1]. Globally 1.1 billion people, including an estimated 638 million in India alone, still practice open defecation [1]. Seven out of ten people who are without improved sanitation live in rural areas. Projections make clear that current progress will fall short of meeting the MDG sanitation target to halve the portion of the population without access to improved sanitation by 2015 [1].

Faced with this challenge, governments, non-governmental organizations (NGOs) and others have undertaken large-scale efforts to expand sanitation coverage. The most ambitious of these is the Governments of India’s Total Sanitation Campaign (TSC), recently revised and renamed the Nirmal Bharat Abhiyan, which was first implemented in 1999 [2]. The TSC is a low-subsidy regime that aims to generate household involvement and demand responsiveness for the building of individual household latrines in below poverty line (BPL) households [3]. It also uses information, education and communication strategy in rural areas designed to generate demand, elicit greater community involvement and encourage use of latrines [4].

The TSC has been largely effective in increasing latrine coverage. According to Government of India records, almost 90 million individual household latrines have been built as a result of the campaign [5]. In addition to the subsidies, the TSC operates a scheme called the Nirmal Gram Puraskar that provides community incentives to Gram Panchayats (local governments) for achieving full open defecation free status [6]. Recent changes under the Nirmal Bharat Abhiyan reforms extend the subsidies beyond BPL households to specified groups. However, most households that are above the poverty line do not qualify for subsidies and must build their own latrines. Perhaps as a result, latrine coverage in villages usually falls well short of 100% [6], [7].

While work continues on achieving sanitation coverage, programme implementers also face the challenge of securing their use by householders. Achieving consistent and widespread use is a common problem for top-down, subsidy-driven sanitation campaigns. It is one impetus for community-led total sanitation, an approach that emphasizes the adverse impact of any non-compliance and uses community-wide mobilization and behaviour change strategies in lieu of subsidies in an effort to achieve lasting open defecation free status [8]. However, securing such compliance is a particular challenge in rural India where open defecation is the norm; two-thirds of the estimated 1.1 billion people who practice open defecation worldwide reside in India [1]. Unlike improved water supplies that are readily embraced in rural settings, achieving latrine use within a population requires changes in private behaviours based on deeply held cultural practices [9]. In a recent assessment of a 5-year water, sanitation and hygiene promotion programme in the southern Indian state of Tamil Nadu, investigators reported a substantial increase in latrine coverage, from 15% to 48%; however, even among households that had built a latrine, 39% of adults and 52% of children were reported to continue the practice of open defecation [10].

Achieving both coverage and use, however, are essential in order to realise the health benefits associated with improved sanitation. Even a comparatively small number of non-users can contaminate the environment with faecal pathogens, causing direct exposure to faecal pathogens through contact and indirect exposure via mechanical vectors (flies) and contaminated drinking water [7], [11]. Microbiological evidence and modeling based on quantitative microbial risk assessment suggests that high levels of coverage and use are necessary to minimize exposure and prevent disease [12]–[14].

Our research group is undertaking a cluster randomized, controlled trial to assess the impact of the TSC as implemented by Water Aid and its NGO partners in a costal district in Orissa (Odisha), a state in Eastern India where open defecation is still widespread and faecal-oral diseases are common [15]. While the study will document the impact of the intervention on latrine coverage and use, it will only follow the population for 21 months following a 12-month implementation period. In order to explore the impact of such an intervention over a longer period, we undertook this cross-sectional study in non-study villages in the same district where the TSC was implemented at least three years previously.

## Methods

### Study area and village selection

The study was conducted in June and July 2012 among 20 villages in Puri District, a rural region located on the coast of the East Indian state of Orissa. Villages were eligible for inclusion in the study if the TSC was undertaken by an implementing partner NGO of WaterAid India at least three years prior to the study. Participating villages were selected randomly from a list of 35 eligible villages provided by implementing partners of WaterAid India.

### Household selection and enrollment

All households in the selected villages were eligible for inclusion in the study. Sampled households were selected randomly following a sampling strategy used for the Extended Program on Immunization (EPI) [16]. A pen was spun in a central location in the village to determine the direction in which the enumerator would sample households. Every second household was sampled until the enumerator reached their quota of households or until they reached the boundary of the village. If the boundary was reached prior to meeting the quota, the enumerator returned to the central location repeat the process. Three enumerators were asked to sample at least seven households per villages, though the aggregate number depended in part on logistics. Households were enrolled if they consented to participate after receiving complete details of the study. Non-consenting households or households where no adult was present at the time of the visit by an enumerator were replaced by the next household on the list.

### Survey tool and procedure

The main study tools consisted of surveys and spot checks of latrines by trained enumerators using Oriya, the local language. Separate surveys for households with and without access to latrines were developed, translated, piloted and back-translated to confirm accuracy. Each survey included questions on basic demographics, size of household, whether the household had a BPL card, type of household construction, religion, highest level of education of female and male heads of household, and distance to nearest water source. They were also asked about exposure to sanitation promotion messages as part of the TSC implementation. Surveys were conducted with the consenting female head of household, or in her absence, a male or female over 18 years.

### Assessing coverage and use

Household latrine coverage was assessed using the question “does your household have a latrine?” Those that answered affirmatively were classified as having a latrine. In households with a latrine, enumerators visually examined the latrine and assessed its functionality [17]–[18]. Latrines were considered “functional” if they met the following criteria: walls over 1.5 meters, some type of closure over the entry for privacy, an unbroken and unblocked toilet pan and a functional pan-pipe-pit connection. Households that had a latrine were asked if the latrine was used by any member of the household. Those that responded affirmatively were further asked to report the age, gender and place of defecation of each member of the household.

### Data Entry and Analysis

Data was entered using EPIData 3.1 and analysed using STATA 12. Bivariate analysis of associations between risk factors and outcome variables was conducted using chi square tests. Logistic regression was then performed to examine the strength of association between covariates with a p value <0.05. To investigate the association between the covariates and latrine coverage and the association between the covariates and latrine use, multivariable models were built using a hierarchical conceptual framework [19]–[20]. To avoid an excess number of variables and unstable estimates in the subsequent model, only variables with a p-value of <0.10 were kept in the subsequent model analysis [20]. In order to adjust for clustering within villages, generalized estimating equations with robust standard errors were used in multivariate analysis.

### Ethics

The study was approved by the ethics committees of the London School of Hygiene and Tropical Medicine and Xavier Institute of Management Bhubaneswar. Surveys and observations were undertaken only after obtaining informed written consent using a prescribed information sheet. No compensation was paid to study participants. In order to ensure anonymity, no names were recorded during data collection and the analysis was done using household codes.

## Results

### Sampled Population

Table 1 provides information on the 20 villages included in the study, including year of TSC implementation. Villages were located within 5 different blocks in the Puri district. Four NGOs had implemented the TSC in the study villages 3 to 8 years prior to our study (mean 5.3 years).

**Table 1:** Village, year of implementation, implementing partner, coverage and use.

Village	Year of TSC Implementation	No. Households Sampled	% Latrine Coverage	% Reported Latrine Use for households and individuals with a latrine	
				Households*	Individuals**
Banakhandi	2007–08	25	64	69	56
Banilo	2007–08	21	95	70	50
Bagalei	2008–2009	26	58	63	47
Begunia	2006–07	25	72	58	43
Nagapur golapur	2006–07	27	48	86	65
Dahangaria	2006	20	55	82	56
Orei***	2006–07	21	90	63	61
Bhanapur	2005	21	86	44	36
Hantapada sasana	2004	22	68	67	59
Panidola	2007	20	60	67	46
Ganeswarpur	2006–07	22	95	90	72
Hatasahi	2006	22	86	74	56
Bantalsingh deuli	2007	22	86	74	69
Swainkera	2007	21	90	47	33
Paridobandha	2007	22	86	26	11
Mathasahi	2007	24	58	13	10
Goudasahi	2007	23	78	56	28
Pradhansahi	2007	18	44	0	0
Baliapatana	2007	24	38	75	21
Tandikera	2008	21	86	89	76
**Total/Mean**		**447**	**72**	**61**	**47**

* Percentage of households that reported at least one member used the latrine sometimes.

** Percentage of household members that were reported to be using the latrine all of the time.

*** Awarded Nirmal Gram Puraskar and open defecation free status.

A total of 447 households were sampled from these 20 villages, representing a mean of 22.5 households sampled per village (range = 18 to 26). This yielded data on 1933 individuals who lived in households that had a latrine. The median number of people per household was 5 (95% CI 5,6) with a range from 1 to 30 people per household (data not shown). The majority of households (68%) either presented a BPL card or claimed to have one. Most (79%) households had heard of a program promoting latrine construction, though fewer (31%) had heard of Village Water and Sanitation Committee (VWSC) members or (20%) had heard of VWSC meetings.

### Latrine coverage and characteristics

Latrine coverage among villages ranged from 38% to 95%, with a median of 75% and a mean of 72% (95% CI = 64,80) (Table 1). In Orei, a village certified as open defecation free, coverage was 90%.

Of the 321 latrines in the study villages, 150 (47%) met the functionality criteria (walls over 1.5 meters, some type of closure over the entry, an unbroken and unblocked pan and a functional pan-pipe-pit connection) (Table 2). More than half (65%) were built with TSC subsidy of cash or materials and most (88%) were pour flush latrines. Few of the latrines sampled had a broken or blocked pan (11%) or non-functional pan-pipe-pit connection (7%), though many (44%) lacked a closure over the entry for privacy.

**Table 2:** Latrine Characteristics.

Covariate	Number (%)
Number of households with latrines	321 (72)
Received cash or materials from NGO for building of latrine	209 (65)
*When the latrine was built*	
Less than 3 years ago	81 (25)
3 to 10 years ago	166 (52)
More than 10 years ago	68 (23)
*Type of latrine*	
Pour flush pit latrine	282 (88)
Direct drop pit latrine	19 (6)
Other	20 (6)
*Height of latrine walls*	
Below 1.5 meters	114 (36)
Over 1.5meters	205 (64)
*Any type of closure over entry for privacy*	
No	142 (44)
Yes	178 (56)
*Any type of roof*	
No	153 (52)
Yes	143 (48)
*Pan condition*	
Broken/Blocked/Choked	32 (11)
Not broken	265 (89)
*Pan-pit pipe connection*	
Not connected	20 (7)
Connected and functional	285 (93)
*Number of pits*	
One	269 (87)
Two	41 (13)
*Pit covering*	
Pit open or mainly open	12 (4)
Pit visible and fully covered or buried	299 (96)
*Size of pit*	
Fewer than 3 rings	15 (5)
3 rings or more	190 (64)
Tank (no rings)	91 (32)
*Number of times pit has been emptied*	
Never	286 (91)
Once or more	29 (9)
*Latrine functional* *	
No	171 (53)
Yes	150 (47)

* Walls over 1.5 meters, some type of closure over the entry, unbroken and unblocked pan and a functional pan-pipe-pit connection.

NGO Non-Governmental Organizations.

In multivariable analysis, the variables that were significantly (p = <0.05) associated with having a latrine were: type of household construction, having heard of a latrine promotion program and having heard of VWSC members (Table 3). Households made of Pucca (concrete) had almost 4 times the odds of having a latrine than Kucha (mud and dung) households (aOR = 3.57 95% CI = 2.25,5.65, p = <0.001). Households who had heard of a program promoting latrine construction (aOR = 2.07 95% CI = 1.17,3.66, p = 0.012) and those who were aware of VWSC members (aOR = 2.07 95% CI = 1.03,4.15, p = 0.04) had more than double the odds of having a latrine than those who had not.

**Table 3:** Multivariable regression analysis of factors associated with latrine coverage.

Coverage Multivariable Analysis				
Covariates	Household with latrine	Adj OR	95% CI	P value (Wald)
*Household construction*				
Kucha	58	1		
Semi-Pucca	67	1.71	1.08,2.73	0.023
Pucca	80	3.57	2.25,5.65	<0.001
*Heard of a program promoting latrines*				
No	57	1		
Yes	75	2.07	1.17,3.66	0.012
*Heard of VWSC members*				
No	66	1		
Yes	85	2.07	1.03,4.15	0.040

Denominators vary as not all respondents answered all questions.

### Latrine use

Of the 126 households (28%) that did not have a latrine, informants reported that all members of the household practice open defecation. Among the 321 households (72%) that had latrines, 62% reported that at least one member of the household was using the latrine (Table 1). However, less than half (47%) of the individuals at these households reported using them all of the time (Table 4). Of these, 54% were females. Even among these households with latrines, 37% of householders were reported to always practice open defecation. Another 5% reported always defecating in the compound; these were mainly young children (Table 4). The remaining individuals were reported to either use the latrine “sometimes” or “usually” (usually was defined as more often than not) (Table 4).

**Table 4:** Reported place of defecation for individuals in households where there is a latrine *N = 1933.*

Place of defecation	Number (%)
Always use a latrine	904 (47)
Usually use a latrine	49 (30)
Sometimes use a latrine	150 (8)
Always open defecation	723 (37)
Always open defecation within the compound	106 (5)

The most common reasons why latrines were not in use was that individuals within households preferred open defecation (29%), the latrine was not complete (28%) or using a latrine was deemed inconvenient (20%). Other reasons for non-use were that the latrines lacked privacy (23%), were used for storage (22%), were broken (17%) or blocked (9%). Only one household ascribed non-use to water being too distant, and only 4% of households reported that it was too difficult to empty the pit.

In the multivariable analysis of latrine use, households that had built their latrines over 10 years ago had more than 4 times the odds of using their latrine (aOR = 4.59 95%CI = 1.82,11.60, p = 0.001) (Table 5). Latrines with walls over 1.5 meters (aOR = 10.21 95% CI = 4.01,26.00, p = <0.001), those with a pan that is not broken (aOR = 8.89 95% CI = 2.56,30.84, p = 0.001) and those with a fully covered pit (aOR = 43.74 95% CI = 4.44,430.70, p = 0.001) were also more likely to be in use. Latrines with any type of closure over the entry (door) were much more likely to be in use (aOR = 42.98 95% CI = 18.13,101.92, p = <0.001) (Table 5). All of the households with a pan pipe-pit connection that did not function were not using their latrine. Latrines which had walls over 1.5 meters, a closure over the entry, an unbroken and unblocked pan and a functioning pan-pipe-pit connection (functional latrines) were more likely to be used than non-functional latrines (aOR = 25.59 95%CI = 12.07,54.26, p = <0.001).

**Table 5:** Multivariable regression analysis of factors associated with latrine use.

Use Multivariable Analysis				
Covariates	Household reporting latrine use	Adj OR	95% CI	P value (Wald)
*When was the latrine built*				
Less than 3 years ago	48	1		
3 to 10 years ago	60	2.54	1.07,6.04	0.034
More than 10 years ago	90	4.59	1.82,11.60	0.001
*Height of latrine walls*				
Below 1.5 meters	30	1		
Over 1.5meters	81	10.21	4.01,26.00	<0.001
*Any type of closure over entry for privacy* **				
No	23	1		
Yes	94	42.98	18.13,101.92	<0.001
*Pan condition*				
Broken/Blocked/Choked	13	1		
Not broken	74	8.89	2.56,30.84	0.001
*Pit covering*				
Pit open or mainly open	8	1		
Pit visible and fully covered or buried	66	43.74	4.44,430.70	0.001
*Latrine Functional* ***				
No	33	1		
Yes	95	25.59	12.07,54.26	<0.001

Denominators vary as not all respondents answered all questions. Use is based on reported use.

** Closure over entry and roof assessed in a model which excluded walls because no latrines without walls had a roof or door.

*** A functional latrine is defined as a latrine which has walls over 1.5 meters, some type of closure over the entry, an unbroken and unblocked pan and a connected and functional pan-pipe-pit connection.

aORs for functional latrines assessed in a model which included village, household construction, pit covering and length of time since latrine has been built.

### Perceived benefits of latrine use

**Figure 1:**
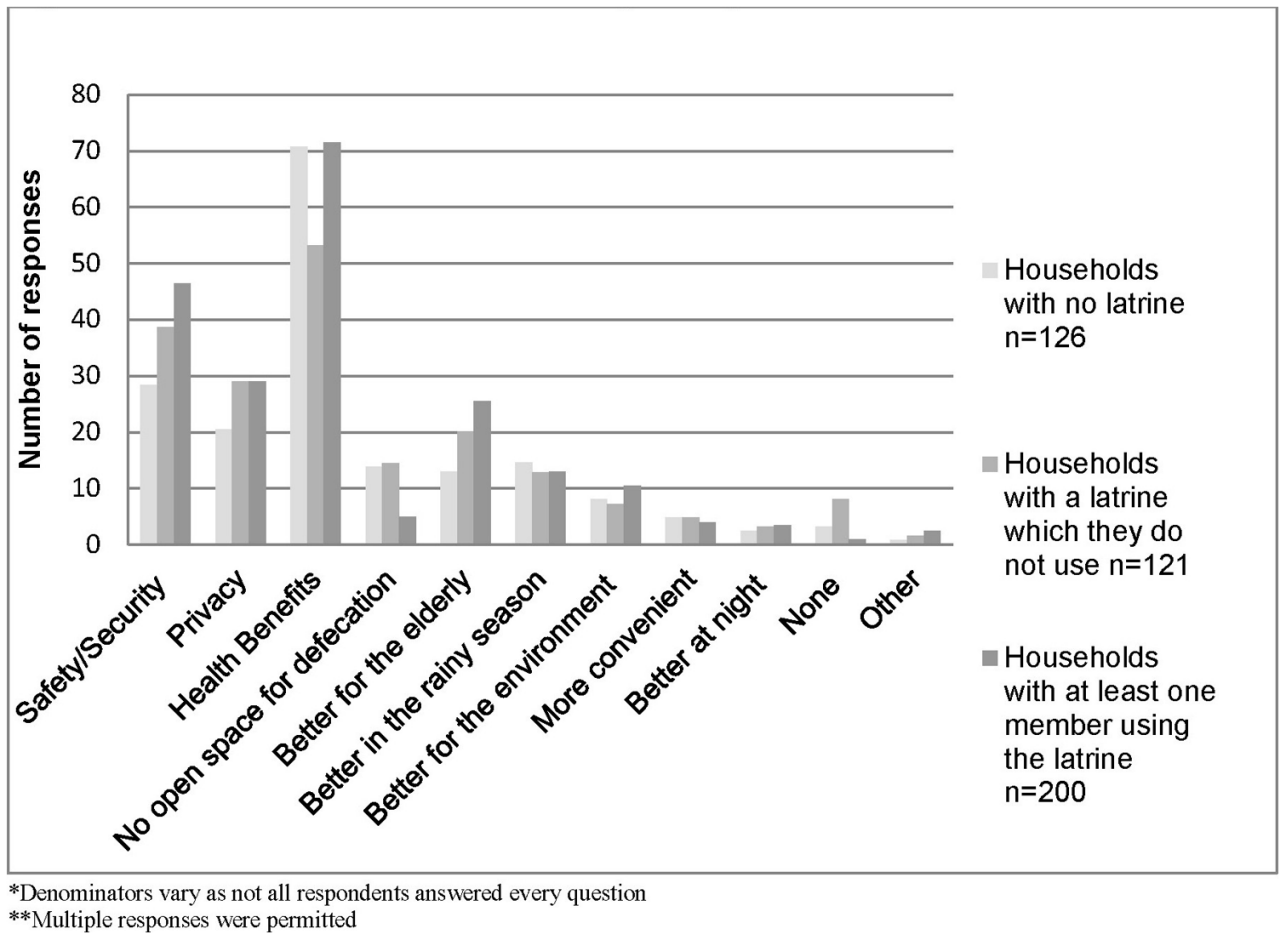
Benefits of latrine use according to respondents. Regardless of whether a household had a latrine, or whether it was in use, the most commonly reported benefit of latrine use was health benefits, followed by safety and security. Households that had a latrine that was in use were less likely to be aware of whether there was no open space for defecation. Few households reported that using latrines were more convenient or better at night.

When asked what the benefits of latrine use were, 66% suggested that there were health benefits associated with latrine use, 39% believed that latrines provided safety and security for women or girls and 27% felt they provided privacy (Figure 1). Of those reporting that there is no open space for defecation, 77% either did not have a latrine or were not using their latrine. No associations were found between the perceived benefits of having a latrine and latrine use.

## Discussion

We undertook a cross-sectional study to assess latrine coverage and use in 20 villages where the TSC had been implemented at least three years previously. If high levels of both coverage and use are necessary to minimize exposure and optimize health impact, our results show deficiencies in both areas.

While the evidence suggests that the campaign was effective in increasing coverage, there were shortcomings. Almost half of the villages achieved at least 80% coverage. While there is no pre-intervention data from these villages, baseline data from a large trial in 100 villages in the same district showed pre-intervention coverage of 8.2% [15]. Given that the TSC extends only to BPLs and limited classes of other priority groups, this suggests that the campaign was effective in significantly increasing latrine coverage among this population. However, coverage was not universal, even in the village with open defecation free status. Moreover, 9 of the 20 villages sampled achieved less than 70% coverage, with 3 reaching less than 50%. This wide variation is consistent with findings from previous studies and demonstrates a need for more consistent implementation of the TSC [6], [7], [21]. There are also issues about the quality or longer-term robustness of the latrines; only 47% met basic criteria established for functionality. Finally, despite targeting the campaign to BPL households, coverage was associated with more costly home construction (pucca rather than kucha); there was also some evidence of an association between latrine construction and secondary education of the female head of household.

Securing consistent use of the latrines represents an even greater challenge. Of the 72% of households sampled that had latrines, more than a third (39%) were not being used by any member of the household. This figure is lower than that reported in similar studies [17], [22], [23] but higher than the 48% reported from Tamil Nadu [10]. Less than half (47%) of householders with access to their own latrines reported always using them for defecation. Consistent with previous research, more women used latrines exclusively than men though the difference (females 54% and males 46%) was not as large as has been seen elsewhere [24]. Well over a third of the members of such households reported never defecating in the latrines; another 8% reported using them only occasionally. Combined with the 28% of households that did not have latrines, it is clear that most defecation events in these communities are still practiced in the open and not in a latrine.

These results suggest that the TSC has not succeeded in substantially reducing exposure to human excreta in these villages. Under these circumstances, it is not clear whether the TSC would be capable of achieving health gains in these communities [7], [11]. Even if only a few members of the community are defecating in the open, the risks to health remain substantially high [12], [14], [25]. This may be particularly true if the refractory members of the community are more likely to be “super shedders” or if safe disposal of child faeces is poor, an important source of exposure [26].

However, the actual impact of sanitation on human health is complex, and the level of coverage and use that is necessary to prevent disease is not well understood [21]. A recent working paper that carefully and comprehensively analyzes datasets on TSC implementation and child health has found the campaign to be associated with significant reductions in child mortality and child stunting [7]. While such study designs are susceptible to unknown confounders and offer more limited potential for causal inference, it is possible that even sub-optimal levels of coverage and use can deliver favorable health outcomes.

The most common reason reported for not using a latrine was that people prefer open defecation. Open defecation is a cultural practice that is deeply engrained in communities in India [27]–[28]. In a study conducted in rural southern India, respondents reported that open defecation did not carry stigma and was hygienically preferable to using a latrine, since they were not accumulating faeces near the house [29]. While the TSC includes social mobilisation and information, education and communication activities that are aimed at overcoming the cultural practice of open defecation within communities [28], [30], our results suggest that this aspect of the campaign may be sub-optimal. If so, this may be a structural deficiency in the TSC, as campaign implementers are compensated for building latrines (coverage) and not for securing their use. New technologies that discretely and objectively monitor latrine use [31] could be incorporated into the TSC in order to compensate programme implementers for securing sustained use. Restructuring the campaign to focus on longer-term use may also address some of the deficiencies in quality and sustainability of construction.

In June 2012, the Government of India revised the TSC and renamed it as Nirmal Bharat Abhiyan. Among other things, the revisions seek to secure 100% coverage in communities. The major revisions of the programme are (i) an increased focus on administration at the Gram Panchayat level, (ii) expansion to include above poverty line households as well as below poverty line households, (iii) an increase in the subsidy with greater flexibility on the latrine type, (iv) inclusion of the schools, and (iv) additional management of the waste stream [2]. This shift in focus was inspired by the reported success of the Nirmal Gram Puraskar aspect of the TSC which provided monetary incentives to achieving open defecation free villages and promoted 100% latrine coverage in rural areas [6]. Our study included one village that had previously been awarded Nirmal Gram Puraskar status. Although coverage was relatively high (90%) in this village, use of latrines was well below optimal at 63%. It is not clear whether the revisions to the programme will be more successful in optimizing latrine use.

However, another reason for low use may be that the latrines are of poor quality. Of the 321 latrines that we sampled, only 150 (47%) met the criteria for functionality, including minimal wall height and a door or other closure to ensure privacy. This is lower than what has been reported in other studies [17], [23]. Functional latrines were much more likely to be used; sufficient wall height, roofs, functional pans, buried or covered pits and doors or other closures to ensure privacy were all associated with higher levels of use. Overall, 95% of ‘functional’ latrines were in use, compared to only 33% of those that were not considered as ‘functional’. On the other hand, latrines that householders wish to use are also more likely to be better constructed and maintained, and lack of latrine use may lead to lack of latrine functionality. The recent revisions to the campaign do not clearly address these construction deficiencies. While the increased subsidies and greater design flexibility may yield higher quality latrines, they may also attract more opportunistic implementers to the sector.

This study has several important limitations. First, like any cross-sectional design, the study offers few insights into temporal relationships between the TSC and latrine ownership and use. Second, the selection of villages included in the study was not random and the results cannot be generalized beyond the 20 villages included in the study. Though the villages were randomly selected from a list provided by the implementing organization, we cannot rule out the potential for selection bias. Third, the EPI sampling strategy has certain limitations [16], and the absence of village census data prevented us from using population proportional sampling or other methods that may have helped ensure the accuracy of our estimates of coverage and use within each community. Fourth, it is also possible that because the study was carried out in rainy season, use of latrines was higher than at other times in the year. There is also the potential for courtesy bias in self-reporting of latrine use [31] however; it is likely that both of these factors would exaggerate the actual level of use, rendering our estimates conservative. Future studies should attempt to use a range of methods to measure use, possibly including instrumented monitoring [31]. Finally, this study provides no evidence of the extent to which various levels of latrine coverage or use impact exposure to faecal pathogens or health outcomes such as diarrhoea, intestinal nematode infection, or stunting. These will be addressed in the trial that is due to be completed in late 2013 15].
